# Kaposi Sarcoma of the Adrenal Gland Resembling Epithelioid Angiosarcoma: A Case Report

**DOI:** 10.1155/2011/898257

**Published:** 2011-08-09

**Authors:** Hassan Huwait, Adam Meneghetti, Torsten O. Nielsen

**Affiliations:** ^1^Anatomical Pathology JP1401, Vancouver Hospital, 855 West 12th Avenue, Vancouver, BC, V5Z 1M9, Canada; ^2^Department of Surgery, 5th Floor Diamond Health Centre, 2775 Laurel Street, Vancouver, BC, V5Z 1M9, Canada

## Abstract

Patients with human immunodeficiency virus infection are known to have increased risk of various neoplasms, including Kaposi sarcoma, which classically involves the skin and mucosal locations. The anaplastic variant of Kaposi sarcoma is rare and poorly documented in the literature. It is characterised clinically by a more aggressive behaviour and increased metastatic potential, and histologically by increased cellularity, mitotic rate, and rarely by epithelioid angiosarcoma-like morphology. We report herein a 64-year-old man with a long-standing history of human immunodeficiency virus infection who developed a right adrenal tumor with a high-grade anaplastic angiosarcoma-like morphology. Immunohistochemistry for human herpes virus-8 was strongly positive in the tumor cells. To the best of our knowledge, this is the first report of an anaplastic Kaposi sarcoma in the adrenal gland.

## 1. Introduction

Kaposi sarcoma (KS) is a rare low-grade malignant vascular neoplasm caused by human herpes virus-8 (HHV-8) and commonly affects the skin of lower extremities, face, trunk, and genitalia. Lymph nodes and other visceral organs are also frequently involved [1]. KS has been reported less frequently in other anatomic locations such as the musculoskeletal system, nervous system, larynx, eye, major salivary glands, and endocrine glands including the adrenal [1–3], heart, breast, and urinary system [1]. Kaposi sarcoma presents in various clinical forms including classic, endemic (African), iatrogenic (transplantation-associated), and AIDS-related KS.

Anaplastic transformation of KS, which is defined histologically as areas of increased cellular pleomorphism and mitosis [4] and clinically as a disease with a more locally aggressive behaviour and metastatic potential, is a known phenomenon, but its exact incidence is indeterminate in the literature. 

We report herein the case of 64-year-old man with a long-standing history of human immunodeficiency virus (HIV), treated with antiviral therapy, who developed a right adrenal mass. Excision revealed a high-grade spindle and epithelioid malignant vascular neoplasm that was positive for both vascular markers and HHV-8 immunostaining, consistent with anaplastic KS. To the best of our knowledge, this is the first case report in the literature of an anaplastic KS resembling angiosarcoma in the adrenal gland.

## 2. Case Report

A 64-year-old man with a long-standing history of HIV diagnosed over 20 years ago, presented in the early 1990s with Kaposi sarcoma of the right and left legs and left hand. He was treated with vinblastine alternating with vincristine and had a good response and control of the disease at that point. Subsequently, the patient had multiple relapses of his cutaneous KS, for which he received different modalities of treatments including radiation therapy, chemotherapy, and updated antiretroviral therapy. He appeared to have a reasonably good response. Apart from KS, his past medical history was also significant for multiple skin cancers including basal cell carcinomas on his head, and leg, and melanoma on his back (all completely excised), Ménière's disease, multiple actinic keratoses, and recurrent cellulitis. In the three years prior to development of the current tumor, the patient received kaletra (lopinavir and ritonavir) and tenofovir; viral load was undetectable (<40 copies/ml) and CD4 count was 220/uL around the time of diagnosis. 

**Figure 1:**
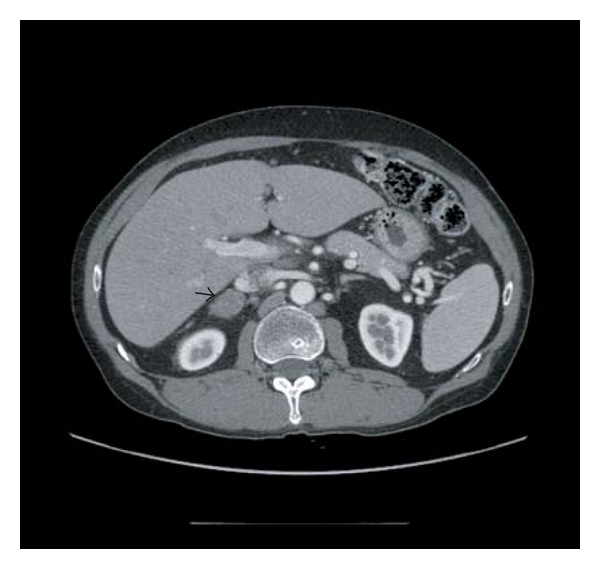
Axial C.T scan image with intravenous contrast. There is an expansile oval mass in the right adrenal gland with soft tissue density (arrow).

The patient subsequently developed cough and had a CT scan of the chest, which disclosed bilateral pleural thickening, and an incidental right adrenal mass lesion, with no other abnormalities detected. The patient was then referred for a core needle biopsy of the right adrenal mass, performed under CT guidance. Following pathological diagnosis (see below), the patient underwent clinical staging via CT scan of the abdomen and pelvis, and a PET scan. As shown in Figure 1, imaging studies characterised a bi-lobed, inhomogeneous mass in the right adrenal gland involving the right adrenal lateral limb and body, measuring 4.6 cm in greatest dimension, with a normal left adrenal gland. The PET scan disclosed a 5.5 cm hypermetabolic focus within the right adrenal gland with no evidence of other hypermetabolic foci elsewhere in the body. An endocrine workup revealed no underlying endocrine abnormalities. The patient was referred for a laparoscopic right adrenalectomy following the core needle biopsy.

## 3. Pathological Findings

The core needle biopsy revealed a mixture of spindle and epithelioid cells admixed with lymphoplasmacytic infiltrate, occasional slit-like vascular spaces, and focal necrosis. Immunohistochemistry was positive for CD31 and HHV-8, and negative for melanoma markers (S-100, and Melan A).

The excision specimen was a 10 × 7 × 3 cm soft tissue mass with unremarkable outer surface. Cut section revealed a hemorrhagic well-delineated mass measuring 7 × 5 × 2.5 cm, variegated in appearance, and showing an admixture of solid tan-coloured areas and hemorrhagic areas with cystic degeneration. No residual adrenal gland tissue was identifiable grossly.

**Figure 2:**
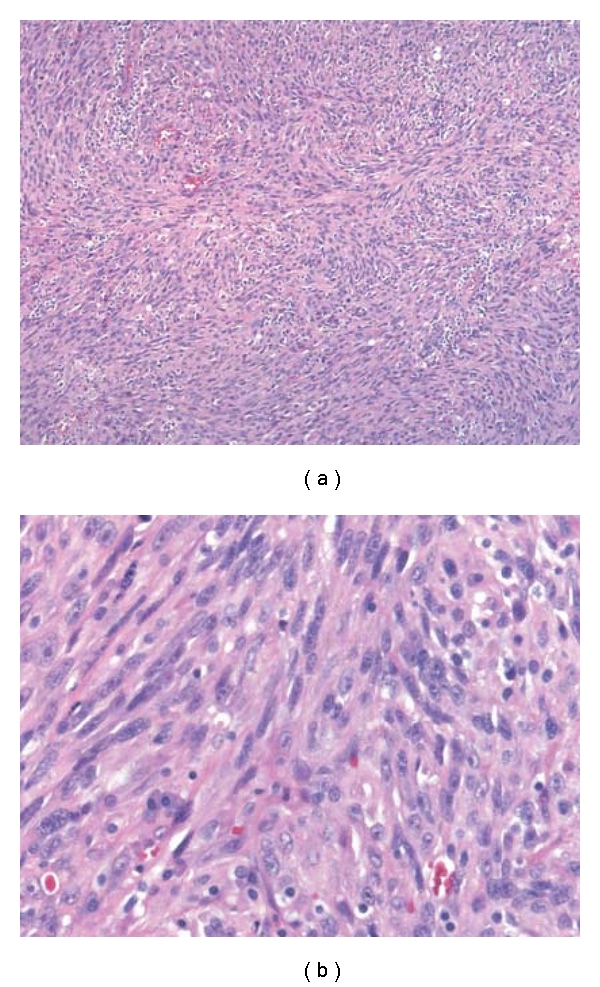
Representative light photomicrographs from the spindle areas of the excision. (a) Low power showing a fascicular growth pattern of spindle cells with occasional slit-like vascular spaces, and lymphoplasmacytic infiltrate. (hematoxylin-eosin, original magnification ×10). (b) Close-up view showing sheets of spindle cells, with few intracytoplasmic hyaline globules and occasional slit-like vascular spaces. (hematoxylin-eosin, original magnification ×40).

**Figure 3:**
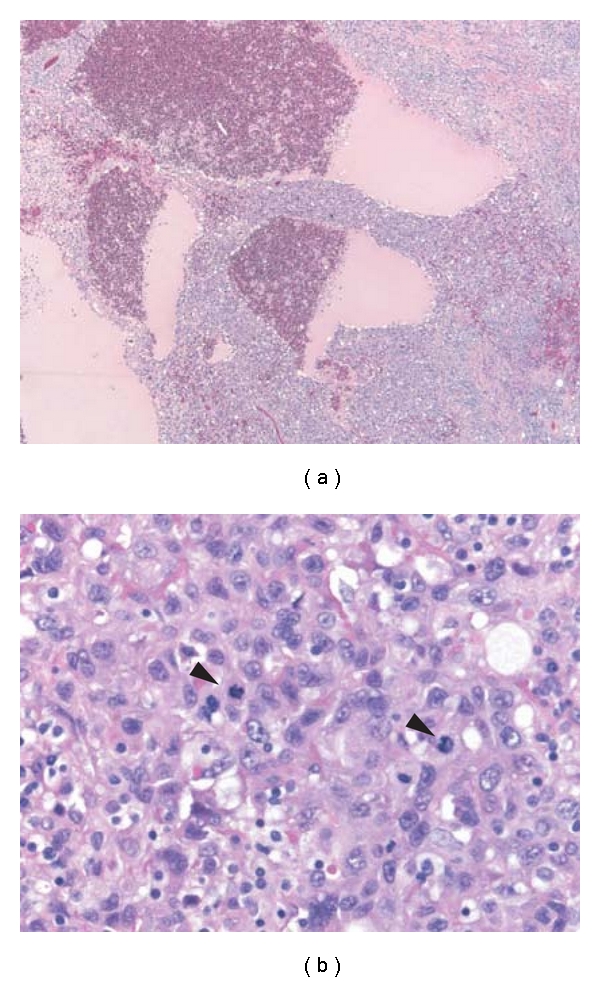
Representative light photomicrographs from the epithelioid areas of the excision. (a) Low power showing large irregular vascular spaces and sheets of epithelioid cells. (b) Close-up view showing large epithelioid cells with moderately abundant cytoplasm, vesicular nuclear chromatin with prominent nucleoli, occasional intracytoplasmic RBC containing lumina, and a high mitotic rate (Arrows).

Multiple formalin-fixed sections of the neoplasm were made and stained with hematoxylin and eosin. Histopathological examination showed total replacement of the adrenal gland by a cellular, pleomorphic and nodular spindle and epithelioid neoplasm. Spindled areas disclosed solid sheets of cells growing in interlacing fascicles and bundles, with a high mitotic count (12/10 high power fields), extravasated red blood cells, occasional slit-like vascular spaces, lymphoplasmacytic infiltrates, and few intracytoplasmic eosinophilic hyaline globules (Figure 2). The epithelioid areas showed a highly pleomorphic atypical epithelioid cells, growing in solid sheets, with large irregular vascular spaces and coagulative tumor-type necrosis. These cells have moderately abundant cytoplasm, large nuclei with vesicular chromatin and prominent eosinophilic nucleoli (Figure 3). Mitotic rate was even higher (20–30/10 hpf). Both intracytoplasmic lumina containing red blood cells and intracytoplasmic eosinophilic hyaline globules were also present focally in these epithelioid areas. 

Immunohistochemistry was performed using standard immunoperoxidase methods: tissue sections were stained with an automated immunostainer (Ventana) following antigen retrieval using an EDTA-based solution (CC1) or a citrate-based solution at pH 6. The primary antibodies used were CD31 (Dako; clone JC70A), CD34 (Cell Marque; clone QBEnd/10), FLI-1 (BD pharmangin; clone G146-254), HHV-8 (Cell Marque; 13B10), D2-40 (Dako; D2-40) S-100 (U of Toronto), MelanA,(Cell Marque; M2-7C10), HMB45 (Dako; clone HMB45), keratin (DakoCytomation), desmin (DakoCytomation; clone D33), smooth muscle actin (DakoCytomation; clone 1A4), and p53 (DakoCytomation; clone DO-7). The primary and secondary antibodies and avidin-enzyme conjugate were visualised using the precipitating enzyme DAB (diaminobenzidine). 

**Figure 4:**
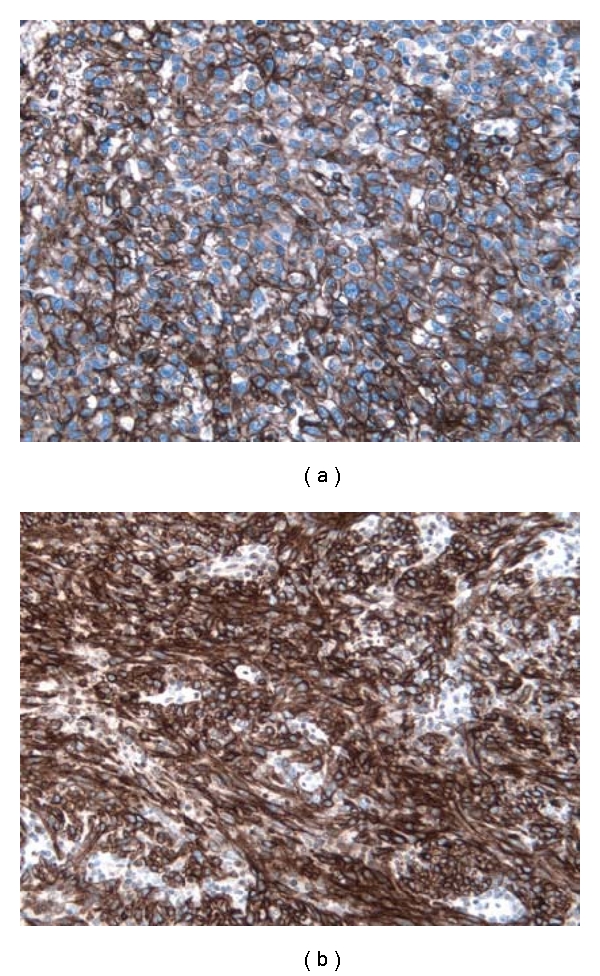
Representative immunohistochemical results for vascular markers. (a) CD31, showing strong and diffuse membranous staining of the endothelial cells. (original magnification ×20). (b) D2-40, showing strong and diffuse membranous staining of the endothelial cells. (original magnification ×20).

**Figure 5:**
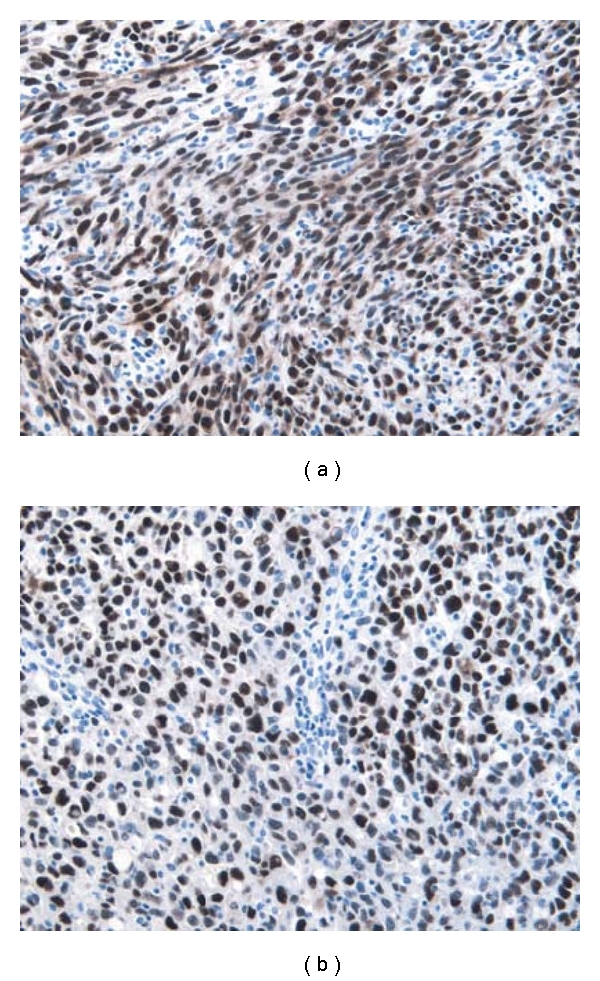
Representative immunohistochemical result for HHV-8, showing moderately intense nuclear staining with a granular pattern for both (a) the spindle and (b) epithelioid cells. (original magnification ×20).

Immunohistochemistry was positive for CD31, CD34, and D2-40 in a strong and diffuse membranous pattern (Figure 4). FLI-1, HHV-8, and p53 showed a strong nuclear staining with a granular pattern for HHV-8 in both the spindled and epithelioid cells (Figure 5). Tumor cells were negative for keratin, S-100, MelanA, HMB45, desmin, and smooth muscle actin.

On the basis of the given clinical history, histological findings and immunohistochemical results, a diagnosis was made of high-grade Kaposi sarcoma, anaplastic variant.

## 4. Discussion

We report herein the case of a 64-year-old male with long standing history of AIDS and previous Kaposi sarcoma, who developed a high-grade anaplastic Kaposi sarcoma of the adrenal gland, demonstrated by both histological features and immunohistochemistry. To the best of our knowledge, this is the first case report in the literature of anaplastic KS of the adrenal gland.

KS is a vascular neoplasm of low malignant potential often occurring in the setting of HIV infection, first described by Kaposi in 1872. Typically, it affects mucocutaneous sites, such as the skin of the lower extremities, face, trunk, genitalia, and oropharyngeal mucosa [1], but is also reported less frequently in other anatomical locations [1]. Clinically KS is classified into four major groups: classic, endemic (African), iatrogenic (transplantation-associated), and AIDS-related [5]. There are no described specific findings for KS on PET scan. Similar to other soft tissue sarcomas where the standard uptake value (SUV) has been demonstrated to correlate with cellularity, tumour grade, mitotic activity, MIB labelling index, and p53 overexpression. No association is seen with p21WAF1 mdm-2, S-phase fraction or ploidy [6].

The histological appearance of these various clinical types of KS does not differ greatly from one group to another. The earliest patch stage of the disease is characterized by proliferation of small slit-like or ectatic vascular spaces, which dissect between dermal collagen, mostly in the upper dermis [4, 5]. These vascular channels are lined by flattened endothelial cells, with a mild inflammatory infiltrate composed mostly oflymphocytes and plasma cells [4, 5]. The more advanced plaque stage is characterized by a diffuse vascular infiltrate occupying most of the dermis. The neoplastic cells are more spindled and tend to form short fascicles [4, 5]. The nodular stage of KS is even more cellular and characterized by proliferation of spindle cells arranged in fascicles. Slit-like spaces containing red blood cells, and intracellular hyaline globules are identified at this stage of the disease. The individual cells are typically monomorphic, and occasional mitosis can be identified following careful search. Inflammatory cells composed predominantly of lymphocytes and plasma cells and hemosiderin deposition are commonly seen toward the periphery of the lesion together with the dilated vascular spaces [4, 5].

Very few cases of KS have been reported in the adrenal gland, or similar unusual visceral locations [1]; most of these cases were reported in AIDS patients.

 Anaplastic KS is a rare and poorly documented variant [4], and has been reported in the context of classic, African, and AIDS-associated KS [4]. These cases, which were first described by Cox and Helwig, are characterized by increased mitosis, and marked cellular pleomorphism [4]. Most of the cases described in the literature are reported in the skin; other less common locations included the liver, spleen, and lymph nodes [7]. These cases tend to retain a spindle cell morphology resembling other high-grade sarcomas [4, 7], with very few cases reported with an epithelioid, angiosarcoma-like morphology [8]. The present case however is unique in that it displays both the spindle and epithelioid anaplastic morphology and arises in an unusual location, the adrenal gland.

The Kaposi sarcoma herpes virus (KSHV), or HHV-8, described by Cheuk et al. in 2004 [9], is considered the causative agent for KS. Furthermore, immunohistochemical detection of HHV-8 Latency-associated Nuclear antigen-1 (LNA-1) is considered very specific and sensitive for KS and helps in differentiating KS from other possible entities in the differential diagnosis [9]. Although some studies have reported HHV-8 positivity by polymerase chain reaction for viral DNA in angiosarcoma [10–12], Cheuk et al., have indicated in their review that this positivity is likely related to contamination by HHV-8 positive passenger lymphocytes, and/or nonspecific amplification [9].

Other variants of KS reported in the literature include: lymphedematous KS, lymphangioma-like KS, lymphangiectatic KS, telangiectatic KS, bullous KS, hyperkeratotic KS, keloidal KS, micronodular KS, pyogenic granuloma-like KS, ecchymotic KS, and intravascular KS [4].

The principal differential diagnoses in the present case included both melanoma (given the patient's previous clinical history) and angiosarcoma (on the basis of the above-mentioned microscopic findings). For melanoma, the histological features of having spindled and epithelioid cells with occasional slit-like and large irregular vascular spaces, lymphoplasmacytic infiltrate, and extravasated red blood cells, bolstered by the immunohistochemical findings of positive vascular markers evident by CD31, CD34, FLI-1, and D2-40, and negative melanoma markers (S100, HMB45, and Melan-A), overwhelmingly favour KS over melanoma. Distinguishing anaplastic KS from angiosarcoma is more difficult to ascertain on the basis of the histological findings alone, and expression of vascular markers is of no help. However, the aforementioned clinical history, as well as positive immunostaining for HHV-8 strongly, supports the diagnosis of anaplastic KS over that of primary adrenal angiosarcoma. 

Some studies have demonstrated that P53 protein mutation is implicated in the pathogenesis and progression, but not initiation of KS [13, 14]. Indeed, p53 mutation was found to be more commonly expressed in cases with a more advanced stage [13, 15, 16] and therefore, was found to be a useful adjunct to identify cases with a potential to have a more aggressive clinical behaviour. Most of these studies were performed on cases with classic, iatrogenic, and AIDS-related KS. On the other hand, p53 mutation has not been assessed before in anaplastic variant KS. 

In summary, we report the 64-year-old man with a long-standing history of HIV who developed a right adrenal anaplastic KS resembling high-grade epithelioid angiosarcoma. To the best of our knowledge, this is the first case report in the literature of an adrenal anaplastic KS resembling epithelioid angiosarcoma.
